# Efficacy of Lanthanum Carbonate and Sevelamer Carbonate as Phosphate Binders in Chronic Kidney Disease—A Comparative Clinical Study

**DOI:** 10.3390/pharmacy11010027

**Published:** 2023-02-02

**Authors:** Parminder Nain, Narendra Nayak, Mary C. Maj, Rohit Kumar Singh, Jaspreet Kaur, Yujin Jeong, Sabyasachi Maity, Reetuparna Nath, Robert H. Hilgers, Shreya Nauhria, Samal Nauhria

**Affiliations:** 1 CT Institute of Pharmaceutical Sciences, Jalandhar 144020, Punjab, India; 2 Department of Microbiology, St. Matthew’s University School of Medicine, Georgetown P.O. Box 30992, Cayman Islands; 3 Department of Biochemistry, St. George’s University School of Medicine, St. George’s, Grenada; 4 MM College of Pharmacy, Maharishi Markandeshwar University, Ambala 133207, Haryana, India; 5 Medical Student Research Institute, Department of Clinical Medicine, American University of Antigua, St. John’s, Antigua and Barbuda; 6 Department of Physiology, Neuroscience and Behavioral Sciences, St. George’s University School of Medicine, St. George’s, Grenada; 7 Department of Education Service, St. George’s University, St. George’s, Grenada; 8 Department of Pharmacology, St. Matthew’s University School of Medicine, Georgetown P.O. Box 30992, Cayman Islands; 9 Department of Psychology, University of Leicester, Leicester LE1 7RH, UK; 10 Department of Pathology, St. Matthew’s University School of Medicine, Georgetown P.O. Box 30992, Cayman Islands

**Keywords:** chronic kidney disease, ESRD, hyperphosphatemia, sevelamer carbonate, lanthanum carbonate

## Abstract

(1) Background: Hyperphosphatemia is correlated with an increased rate of mortality and morbidity due to cardiovascular diseases in chronic kidney disease (CKD) patients. It can be improved by restricting dietary intake of phosphate and oral phosphate binders, such as lanthanum carbonate and sevelamer carbonate. (2) Objective: To evaluate the clinical efficacy of sevelamer carbonate in comparison to lanthanum carbonate as phosphate binders for the treatment of hyperphosphatemia in CKD patients. (3) Methods: A randomized control comparative clinical study was conducted for one year on 150 CKD patients associated with hyperphosphatemia, divided into two groups, i.e., Group 1 (*n* = 75) treated with sevelamer carbonate 800 mg thrice daily and Group 2 (*n* = 75) treated with lanthanum carbonate 500 mg thrice daily. The patients were assessed at the time of enrollment in the study, after three months and after six months from baseline for different parameters, i.e., complete blood count, liver function tests, renal function tests, electrolytes, and serum phosphate level. (4) Results: 150 CKD patients aged 51–60 participated in the study. The mean age of patients was 54 ± 4.6 years, and males (55.71%) were more common than females (44.29%). Hypertension was the common comorbidity in both groups with chronic kidney disease. After six months of treatment, the mean serum phosphate level was significantly decreased from 8.31 ± 0.09 mg/dL to 5.11 ± 0.18 (38%) in Group 1 and from 8.79 ± 0.28 mg/dl to 4.02 ± 0.12 (54%; *p* < 0.05) in Group 2, respectively. In both groups, no significant difference was found in other parameters such as parathyroid hormone, calcium, uric acid, LFT, RFT, CBC, etc. (5) Conclusion: Lanthanum carbonate is more efficacious in lowering serum phosphate concentrations and effectively managing hyperphosphatemia as compared to sevelamer carbonate.

## 1. Introduction

Chronic kidney disease (CKD) is defined as a gradual loss of kidney function characterized by reduced glomerular filtration rate (GFR) under 60 mL/min/1.73 m^2^ for a minimum of three or more than three months [1]. CKD is related to end-stage renal disease (ESRD) with GFR of less than 15 mL/min representing the end stage of CKD. GFR is one of the most commonly recognized criteria to evaluate kidney function. Albuminuria, present in many patients with underlying diseases such as diabetes and hypertension, increases the risk of CKD and its progression toward ESRD [2]. The National Kidney Foundation Kidney Disease Outcomes Quality Initiative (NKF-KDOQI) divided CKD into five stages and three phases per the albuminuria range [3]. As per World Health Organization (WHO) Global Burden of Disease epidemiology data, CKD was ranked 27th on the list of mortalities worldwide in 1990 but rose to 18th in 2010 [4]. In 2017, there were more than 700 million worldwide cases of CKD. Almost a third of patients with CKD lived in two countries—China (more than 135 million) and India (more than 120 million). CKD contributes to nearly 850,000 deaths per year. Globally, the number of patients requiring renal replacement therapy is projected to cross 5 million by 2030 [5]. A higher CKD burden has been noted, especially in lower- and lower-middle-income countries [6].

The expected deaths due to CKD in India have risen from 3.78 million (4% of all deaths) in 1990 to 7.63 million (66.7% of all deaths) in 2020 [7]. In India, the prevalence is 800 per million for CKD and nearly 150 to 200 per million for ESRD [5,8]. The most common cause of CKD in the clinical setting is hypertension and diabetic nephropathy [9].

Hyperphosphatemia is independently associated with increased cardiovascular complications and mortality in patients with CKD stages 4 and 5 due to impaired renal phosphate excretion. Increased serum phosphate level (above 6.5 mg/dL) has been reported to contribute to an 18–39% higher mortality in CKD patients [10]. Therefore, serum phosphate levels are recommended to be maintained within the reference range (2.5 to 4.5 mg/dL) among other laboratory examinations such as serum calcium and parathyroid hormone levels for the treatment of mineral and bone disorder (MBD) and hyperphosphatemia in CKD [11]. Clinically, phosphate excretion with dialysis and dietary phosphate restriction is performed to manage serum phosphate levels of CKD patients. In addition, phosphate absorption from the gastrointestinal (GI) tract needs to be reduced by using oral phosphate binders [12]. Therefore, pharmacotherapeutic agents such as phosphate binders are commonly prescribed in conjunction to prevent and manage hyperphosphatemia [13]. These agents bind dietary phosphate within the GI tract to inhibit its absorption and increase excretion. Particularly aluminum- and calcium-free phosphate binders such as sevelamer carbonate and lanthanum carbonate are available on the market as treatment options for hyperphosphatemia. Both decrease serum phosphate levels and low-density lipoprotein (LDL) cholesterol and increase high-density lipoprotein (HDL) [14].

The current study aims to investigate the clinical efficacy and safety between sevelamer carbonate and lanthanum carbonate as a phosphate binder in CKD patients to treat hyperphosphatemia.

## 2. Materials and Methods

Study objective, duration, and design: A randomized control comparative clinical study was performed to find the efficacy and safety between lanthanum carbonate and sevelamer carbonate as phosphate binders in CKD patients at a teaching hospital for a time period of one year (August 2016 to July 2017). A total of one hundred fifty (150) CKD patients were enrolled in the study after full screening for present illness, past medical history, and inclusion and exclusion criteria. The current study was approved by Institutional Ethical Committee (MMDU/MMIMSR/IEC/859) and was performed in good clinical practice and per the declaration of Helsinki. The aim and objectives of the study were explained to the patients or their guardians. All patients signed written consent in their preferred language after explaining the study.

Participant inclusion and exclusion criteria: Patients aged 51 to 60 years of both genders were included in the study. The patient should be diagnosed with CKD stage 3–5 (according to the GFR (Cockcroft-Gault method)), with or without concomitant illness. The patients of the geriatric population (above 60 because they are more vulnerable and high prevalence of comorbid conditions), pediatric/children population (below 18), pregnant and lactating women, terminally ill patients (patients with malignancy or having positive HIV, HCN, HCV, or HbsAg), and psychiatric patients were excluded from the study.

Data collection: All the CKD patients in this study were taken from the Inpatient Department and came to the hospital with renal problems. Patients were equally divided into two groups: Group 1 (*n* = 75) was treated with sevelamer carbonate 800 mg thrice daily with a meal (as per recommendations if serum phosphate level is 5.5 to 7.5 mg/dL) immediately after a meal (Film-coated tablet: swallow the tablet whole, do not crush, chew, or split the tablet) and Group 2 (*n* = 75) was treated with lanthanum carbonate 500 mg thrice daily with a meal (Chewable tablet: Chew or crush tablet completely; do not swallow whole). The patients were assessed three times during the six months period, i.e., at the time of patient enrollment in the study (1st visit), after three months from baseline (2nd visit), and after six months from baseline (3rd visit) for different parameters, i.e., complete blood count (CBC), renal function test (RFT), liver function test (LFT), serum electrolytes, parathyroid hormone (PTH), vitamin D, and triglycerides (TG). Blood pressure, pulse rate, respiratory rate, temperature, and body weight were also monitored during this study. Patients were treated according to the protocol during the entire study period.

Data processing and analysis: The Statistical Package for Social Sciences (SPSS v15.0) was used to analyze the collected data. A preliminary inspection of the numerical data included minimum, maximum, mean value, and standard error mean (SEM). The statistical evaluation included independent samples *t*-tests for mean differences concerning laboratory parameters, i.e., CBC, RFT, and serum electrolytes between the first visit and after six months of patients, as well as between Group 1 and Group 2. Results were expressed as SEM and also represented in a percentage manner. The significance level was established at a *p*-value of less than 0.05 (considered statistically significant).

## 3. Results

Based on age, the number of patients in both Groups 1 and 2 was in the range of 51–60 years of age 54% versus 57%, respectively. The percentage of males was higher in both the groups (54% in Group 1 and 57% in Group 2) than the percentage of female patients. Most patients were classified as stage 4 CKD (Table 1).

**Figure 1:**
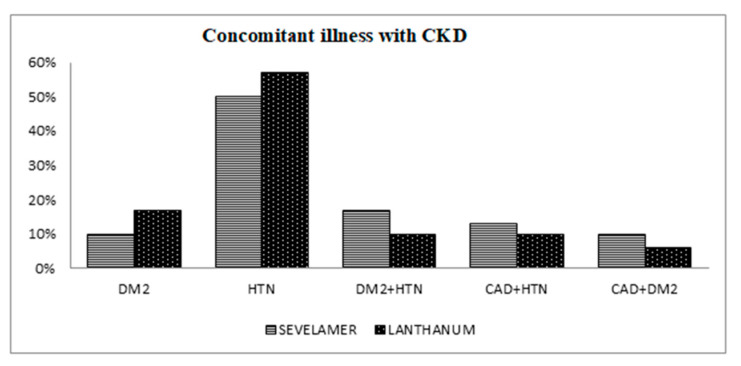
Distribution of patients based on concomitant illness with chronic kidney disease (Results showed in percentage) (CAD: Coronary Artery Disease, DM-2: Diabetes Mellitus-2, HTN: Hypertension).

Both groups were equally divided with regards to the distribution of concomitant illnesses, such as diabetes, hypertension, diabetes with hypertension, coronary artery disease with hypertension, and coronary artery disease with diabetes (Figure 1).

The highest comorbidity in Groups 1 and 2 was found to be hypertension (50% and 57%, respectively) (Figure 1).

Some parameters have shown statistically significant changes from first visit (baseline) to third visit (after six months) in both groups. For example, CBC parameters show a statistically significant increase (*p* < 0.05) in both groups after six months of treatment (Table 2).

After six months of treatment, liver function tests (serum alkaline phosphatase, SGOT, and SGPT), total protein, and serum albumin were found to be unchanged compared to the baseline (Table 3).

Serum electrolytes (Ca^2+,^ Na^+^, K^+^, and Cl^−^) were not statistically significantly altered after 6 months irrespective of treatment (Table 4).

Parathyroid hormone (PTH), vitamin D, and triglycerides levels did not statistically significantly change in both groups (Table 5).

**Figure 2:**
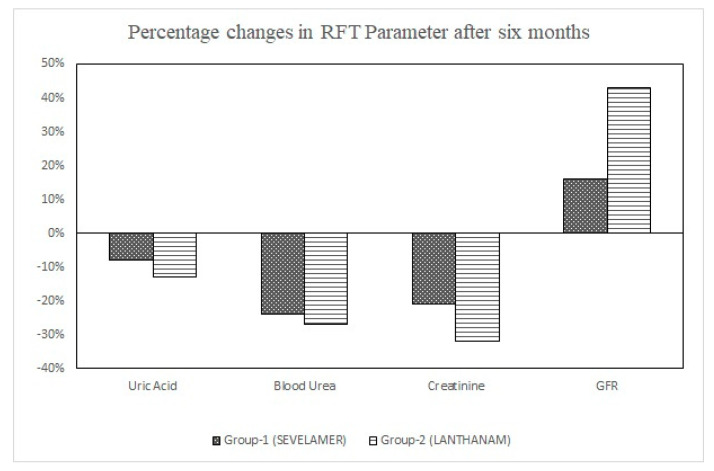
Percentage (%) changes in RFT parameters after six months of treatment.

Sevelamer carbonate affected renal function parameters in many ways. It decreased uric acid (8%), blood urea (24%), and serum creatinine (21%), and increased GFR (from 11 ± 2 to 13 ± 2; 16%). However, none of these changes reached statistical significance. Lanthanum carbonate showed non-statistically significant decreases in uric acid (13%), blood urea (27%), and serum creatinine (32%), but GFR statistically significantly increased (from 10 ± 2 to 17 ± 2; 40%) at the end of the study when compared to baseline (Figure 2).

**Figure 3:**
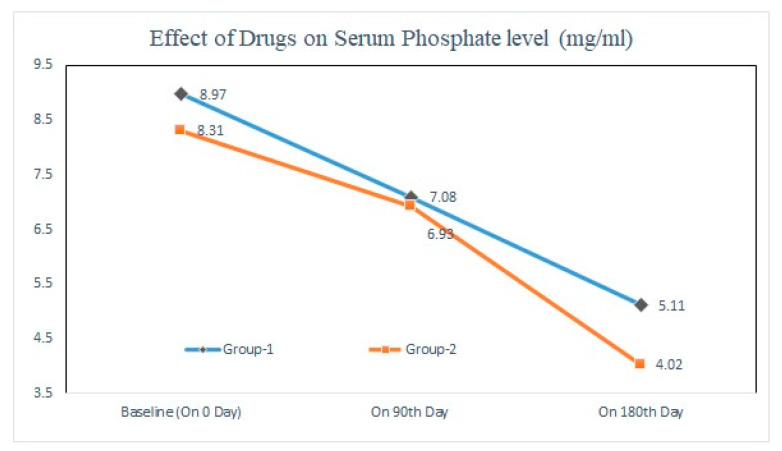
Effect of Sevelamer Carbonate and Lanthanum Carbonate on Serum Phosphate level (mg/mL) at baseline, on 60th day and 90th day in stage III & IV patients.

The results showed that when CKD patients (Stages III and IV) were treated with sevelamer carbonate (Group 1; *n* = 67) and lanthanum carbonate (Group 2; *n* = 64), the level of serum phosphate was statistically significantly (*p* < 0.05) reduced after six months when compared to baseline (in Group 1 from 8.31 ± 2.67 mg/dL to 5.11 ± 1.49 mg/dL; in Group 2 from 8.79 ± 2.11 mg/dL to 4.02 ± 1.30 mg/dL). This drop in serum phosphate concentration was more with lanthanum carbonate as compared to sevelamer carbonate in Stage III and IV patients (Figure 3).

**Figure 4:**
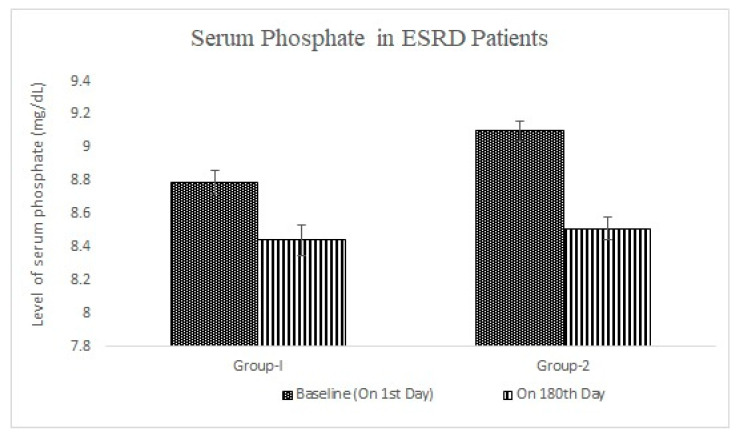
Effect of Sevelamer Carbonate (Group-1) and Lanthanum Carbonate (Group-2) on Serum Phosphate level (mg/mL) in ESRD patients.

The level of serum phosphate was also not statistically significant (*p* < 0.05), and decreased from (8.79 ±0.07 mg/dL to 8.44 ± 0.06 mg/dL) in group 1 (*n* = 8) and (9.1 ± 0.09 mg/dL to 8.51 ± 0.07 mg/dL) in group 2 (*n* = 11) after six months when compared to baseline in ESRD patients (Stage V) (Figure 4).

## 4. Discussion

Phosphate retention eventually leads to hyperphosphatemia, which is associated with poor clinical outcomes and may affect the prognosis in CKD patients [15]. Thus, phosphate control is crucial to prevent mineral and bone disorder in chronic kidney disease (CKD-MBD) management. Results of this study demonstrated that sevelamer carbonate and lanthanum carbonate both were efficacious in control of serum phosphate level, which positively alters other vital organ parameters and are well tolerated in patients who had CKD. Our study concludes that lanthanum carbonate is more efficacious when compared with sevelamer carbonate in the reduction in serum levels of phosphate. Although lanthanum carbonate is a therapeutically more effective drug, it is not the drug of choice for medical practitioners as well as patients because of its metallic taste (as chewable tablets) and high cost [16]. A normal GFR ranges from 120 to 130 mL/min/1.73 m^2^ but it decreases with age [17]. GFR declines by 10 mL/min/1.73 m^2^ for every 10 years after the age above 40 years, in a way that by the age of 70 years, the GFR has declined by nearly 30 mL/min/1.73 m^2^. CKD is characterized by a reduced absolute eGFR < 60 mL/min/1.73 m^2^ for a minimum of 3 months and abnormal renal markers. The National Health and Nutrition Examination (NHANE) survey reported that at the age of 60 years or more, the prevalence of CKD has been increased from nearly 19% to 25% [18].

In a worldwide survey of ESRD, men have a higher incidence and prevalence of CKD than women [19]. Similarly, in the present study, the distribution of patients based on gender in both the groups showed more males than female patients. The principal driving components behind CKD are the increasingly aged global population, type 2 diabetes mellitus, and cardiovascular disease [1]. In our study, the highest comorbid conditions were hypertension and diabetes.

Hyperphosphatemia can be defined as an electrolyte disturbance, i.e., abnormally elevated phosphate levels in the blood because a glomerular filtration rate of less than 30 mL/min significantly reduces the filtration of inorganic phosphate, increasing its serum level. The average adult phosphorus levels should be between 2.5 mg/dL and 4.5 mg/dL. Other less common causes include a high intake of phosphorus or increased renal reabsorption [20].

Phosphorus is a common anion that plays an essential role in energy generation. Through feedback control mechanisms, various organs and glands regulate serum phosphate levels. These include the parathyroid gland, kidneys, and bones. Regulation involves the maintenance of phosphorus in the narrow physiological range. Phosphorus levels in the body are also regulated by fibrogenic growth factor 23 (FGF-23) [21,22]. Phosphate is less soluble at alkaline versus acidic pH, so phosphate precipitates onto the insoluble ammonium products, yielding a large staghorn stone formation. Globally, approximately 80% of kidney stones are composed of calcium oxalate (CaOx) mixed with calcium phosphate (CaP). Hypocalcemia usually occurs because of hyperphosphatemia and phosphate binding with the calcium in tissues. Hypocalcemia increases PTH levels (secondary hyperparathyroidism), resulting in increased bone resorption of calcium, thereby causing soft tissue mineralization and bone de-mineralization. Aluminum- and calcium-based phosphate binders, sevelamer carbonate, and lanthanum carbonate are most frequently used for treating hyperphosphatemia [14,23,24]. In this clinical study, researchers aimed to compare the therapeutic and other vital parameter effects of aluminum- and calcium-free phosphate binder for six months in CKD patients.

CKD-induced anemia is based on multiple mechanisms such as folate (vitamin B12) and iron deficiency, GI tract bleeding, severe hyperparathyroidism, shortened red blood cell survival rate, systemic inflammation, and decreased erythropoietin synthesis in response to renal hypoxia [25,26]. Interstitial fibroblasts secrete a glycoprotein called erythropoietin which is essential for the differentiation and growth of red blood cells in the bone marrow [27]. In a previous study, no evidence of iron accumulation was found in patients with end-stage renal disease receiving lanthanum carbonate [28]. Results from that study reported that the experimental drugs after six years of treatment had no significant effects on hematological parameters. Moreover, the study reported a non-significant effect on hemoglobin and platelet count. A minor improvement in Hb and platelets could perhaps be due to the addition of iron and erythropoietin therapy in patients with chronic kidney disease. Similarly, the current study showed a non-significant effect of experimental drugs in both groups.

The results of the present study indicate the significant increase in eGFR only by lanthanum carbonate. This data supported the fact that there was a significant linear relationship between creatinine and serum urea in CKD. Phosphorus concentrations above 6.4 mg/dL were associated with an increased risk of all-cause mortality [29]. The therapeutic effect of both the experimental drugs in the management of hyperphosphatemia in CKD patients has been demonstrated in several clinical studies, but this study compared the phosphorus level reduction effect in patients with different stages of CKD for the first time. The results of the present study show that both sevelamer carbonate and lanthanum carbonate were effective phosphate binders, as phosphate levels decreased only in stage 3 and stage 4 of CKD, but were not significantly effective in ESRD patients after six months of treatment.

As expected, the decrease in serum phosphorous prompted a decrease in PTH. However, this decline was anticipated since serum phosphorus is known to regulate PTH secretion. Our results demonstrate that PTH levels decreased more during the treatment with lanthanum carbonate as compared to sevelamer carbonate but it was not statistically significant. In addition, experimental studies have demonstrated that long-term lanthanum carbonate treatment inhibited serum PTH and led to renal osteodystrophy [30,31]. These results indicate that lanthanum carbonate can potentially prevent the progression of mineral and bone disorders through the inhibition of phosphate overload. Because phosphate overload is located upstream of all disorders of bone and mineral metabolism in patients with renal failure, its control by phosphate binders is probably the most important treatment strategy [32].

In our study, serum calcium level was significantly increased in both the groups after six months of treatment. There are two likely explanations for this phenomenon. First, since these drugs compete with dietary calcium for phosphate binding, more free calcium may be absorbed because of treatment. Additionally, due to the reduction in serum phosphate level, a reduction in calcium phosphate deposited in tissues occurs, eventually raising the serum calcium concentration [33]. The hypercalcemia observed with lanthanum carbonate treatment is hypothesized from the fact that phosphorus reduction raises calcium levels via increasing calcemic action of PTH [34]. After being produced in the skin, vitamin D activates in the liver and kidneys, and calcitriol is produced. Calcitriol further helps increase the amount of calcium in the bloodstream by absorbing intestinal calcium and prevents calcium loss from the kidneys [35]. In our study, the calcium level is within the normal range after six months, so calcification-associated problems are not clinically observed. Moreover, the level of vitamin D was not significantly changed in both study groups. The effect of sevelamer carbonate and lanthanum carbonate on bone turnover is limited [36,37]. CKD with or without nephrotic syndrome is accompanied by lipoprotein metabolism abnormalities [38]. Experimental and clinical studies have suggested a strong correlation between dyslipidemia and the progression of renal disease. However, the mechanism is unclear, but data suggest that oxidative stress and insulin resistance may potentiate lipid-induced renal damage [39,40]. Several studies reported that sevelamer treatment reduced total cholesterol and LDL but did not affect triglycerides levels [41]. On the other hand, lanthanum carbonate decreases triglycerides in long-term treatment [42]. In our study, lanthanum carbonate reduced triglycerides (only 10%; not statistically significant) compared to the baseline. However, sevelamer carbonate reduced triglycerides (only 2%, i.e., negligible (not statistically significant)). The cholesterol-lowering effect of sevelamer carbonate is probably caused by bile acid binding [43]. Increased synthesis of bile acids results from increased fecal excretion of the same. An increase in bile acid level triggers the up-regulation of LDL receptors leading to a lower systemic LDL level. Lipid-lowering drugs such as cholestyramine and colestipol have similar actions [44].

A limitation of the current study is a limited sample size and data collected from a single center.

## 5. Conclusions

Phosphate retention eventually leads to hyperphosphatemia, which is associated with poor clinical outcomes and may affect the prognosis in CKD patients. Thus, phosphate management is an integral part for mineral and bone disorders in CKD. Results of this study demonstrated that sevelamer carbonate and lanthanum carbonate were both useful in controlling the serum phosphorus in CKD patients. Our study concludes that lanthanum carbonate is more efficacious when compared with sevelamer carbonate. It is not the drug of choice for medical practitioners and patients because of its metallic taste (as a chewable tablet) and high cost. Thus, more effective and targeted treatment and prevention regimes are urgently needed, especially in regions with a high CKD burden.

## Figures and Tables

**Table 1:** Demographic data of patients.

Gender (Sex)	Group 1 n (%)	Group 2 n (%)
Male	41 (54%)	43 (57%)
Female	34 (46%)	32 (43%)
**Age**		
21–35	13 (17%)	11 (15%)
36–50	22 (29%)	22 (29%)
51–60	40 (54%)	42 (56%)
**Area**		
Rural	39 (52%)	42 (56%)
Urban	36 (48%)	33 (44%)
**Stages of CKD**		
Stage 3	28 (37%)	24 (32%)
Stage 4	39 (52%)	40 (54%)
Stage 5	8 (11%)	11 (14%)

**Table 2:** Effect of sevelamer carbonate and lanthanum carbonate on complete blood count level.

Laboratory Parameters	Group 1		Group 2	
	On 1st Visit	After 6 Months	On 1st Visit	After 6 Months
Hemoglobin (g/dL)	8.91 ± 0.39	10.93 ± 0.42	9.06 ± 0.80	10.74 ± 0.50
Total Leukocyte Count (/cumm)	9.16 ± 0.53	9.79 ± 0.36	10.1 ± 0.66	12.11 ± 0.47
Platelet Count (/cumm)	188 ± 13	218 ± 14	198 ± 19	220 ± 20

Results are expressed as mean ± SEM, when compared to baseline. *p* < 0.05 when statistically significant.

**Table 3:** Effect of sevelamer carbonate and lanthanum carbonate on LFT parameters.

LFT	**Laboratory Parameters**	**Group 1 (*n* = 75)**		**Group 2 (*n* = 75)**	
		**On 1st Visit**	**After 6 Months**	**On 1st Visit**	**After 6 Months**
	Alkaline Phosphatase (U/L)	155.16 ± 12.93	109.86 ± 10.69	187.16 ± 34.21	170.8 ± 25.39
	SGOT (U/L)	50.03 ± 13.31	42.25 ± 7.25	41.25 ± 10.71	35.17 ± 4.7
	SGPT (U/L)	40.97 ± 4.10	36.97 ± 4.28	35.58 ± 8.16	32.92 ± 4.76
	Total Protein (g/dL)	6.87 ± 0.17	7.11 ± 0.17	6.48 ± 0.37	6.73 ± 0.16
	Serum Albumin (g/dL)	2.61 ± 0.12	3.86 ± 0.11	2.98 ± 0.23	3.73 ± 0.18

Results are expressed as mean ± SEM.

**Table 4:** Effect of sevelamer carbonate and lanthanum carbonate on serum electrolyte level.

Serum Electrolyte	**Laboratory Parameters**	**Group 1 (*n* = 75)**		**Group 2 (*n* = 75)**	
		**On 1st Visit**	**After 6 Months**	**On 1st Visit**	**After 6 Months**
	Serum calcium (mg/dL)	8.2 ± 0.2	9.5 ± 0.2	8.6 ± 0.2	10.7 ± 0.1
	Serum sodium (mEq/L)	135 ± 1	131 ± 0	139 ± 2	136 ± 1
	Serum potassium (mEq/L)	4.6 ± 0.2	4.8 ± 0.2	4.8 ± 0.3	4.8 ± 0.2
	Serum chloride (mEq/L)	111 ± 1	103 ± 1	109 ± 1	103 ± 1

Results are expressed as mean ± SEM.

**Table 5:** Effect of sevelamer carbonate and lanthanum carbonate on triglycerides/PTH/vitamin D level.

Others	**Laboratory Parameters**	**Group 1 (*n* = 75)**		**Group 2 (*n* = 75)**	
		**On Baseline**	**After 6 Months**	**On Baseline**	**After 6 Months**
	Triglycerides (mg/dL)	180 ± 18	166 ± 15	185 ± 13	157 ± 13
	PTH (pg/mL)	70 ± 3	69 ± 3	72 ± 5	68 ± 5
	Vitamin D (ng/mL)	20 ± 0	23 ± 1	21 ± 1	25 ± 0

Results are expressed as mean ± SEM; when compared to baseline. *p* < 0.05 when statistically significant.

## Data Availability

Not applicable.
